# Influenza virus‐like particles harboring H9N2 HA and NA proteins induce a protective immune response in chicken

**DOI:** 10.1111/irv.12472

**Published:** 2017-08-24

**Authors:** Xin Li, Houbin Ju, Jian Liu, Dequan Yang, Xinyong Qi, Xianchao Yang, Yafeng Qiu, Jie Zheng, Feifei Ge, Jinping Zhou

**Affiliations:** ^1^ Veterinary disease diagnostic center Shanghai Animal Disease Control Center Shanghai China; ^2^ Shanghai Veterinary Research Institute Chinese Academy of Agricultural Sciences Shanghai China; ^3^ College of Biological Sciences and Biotechnology Yangzhou University Yangzhou China

**Keywords:** chickens, H9N2, vaccine, virus‐like particles

## Abstract

**Background:**

Avian influenza viruses represent a growing threat of an influenza pandemic. The co‐circulation of multiple H9N2 genotypes over the past decade has been replaced by one predominant genotype—G57 genotype, which displays a changed antigenicity and improved adaptability in chickens. Effective H9N2 subtype avian influenza virus vaccines for poultry are urgently needed.

**Objective:**

In this study, we constructed H9N2 subtype avian influenza virus‐like particle (VLP) and evaluated its protective efficacy in specific pathogen‐free (SPF) chickens to lay the foundation for developing an effective vaccine against influenza viruses.

**Methods:**

Expression of influenza proteins in VLPs was confirmed by Western blot, hemagglutination inhibition (HI), and neuraminidase inhibition (NI). The morphology was observed by electron microscopy. A group of 15 three‐week‐old SPF chickens was divided into three subgroups of five chickens immunized with VLP, commercial vaccine, and PBS. Challenge study was performed to evaluate efficacy of VLP vaccine.

**Results and Conclusions:**

The hemagglutinin (HA) and neuraminidase (NA) proteins were co‐expressed in the infected cells, self‐assembled, and were released into the culture medium in the form of VLPs of diameter ~80 nm. The VLPs exhibited some functional characteristics of a full influenza virus, including hemagglutination and neuraminidase activity. In SPF chickens, the VLPs elicited serum antibodies specific for H9N2 and induced a higher HI titer (as detected by a homologous antigen) than did a commercial H9N2 vaccine (A/chicken/Shanghai/F/1998). Viral shedding from VLP vaccine subgroup was reduced compared with commercial vaccine subgroup and control subgroup.

## 1 INTRODUCTION

The avian H9N2 influenza virus has been endemic among domestic poultry in Asia since the early 1990s.^1^, ^2^ It readily infects both chicken and duck, as well as the minor poultry species quail, pheasant, partridge, pigeon, silky chicken and chukar.^3^, ^4^ In 1999, 2003, from 2007 to 2009, and 2013 human infection with H9N2 occurred in Hong Kong, Shenzhen, and Hunan, China.^5^, ^6^, ^7^, ^8^ Recently, WHO have reported that three human cases of H9N2 infections have been identified in China from September 27, 2016, to February 27, 2017. Its occasional transmission to humans has raised the possibility of a damaging pandemic spreading through an immunologically naive population.^9^, ^10^

The conventional production of avian influenza vaccines is complicated by the need for a large number of embryonated chicken eggs and disposal facilities. Studies of virus‐like particles (VLPs) in animals and humans indicate that an influenza vaccine platform designed to present both full‐length HA and NA antigens in VLP form may better emulate native presentation of these antigens to the immune system, yet at the same time avoid the safety concerns posed by a live, replicating agent.^11^ Moreover, it was reported that the heterologous co‐expression of the H3N2 genes HA, NA, M1, and M2 in insect cells has been shown to result in the self‐assembly of VLPs, which act protectively against H3N2 in mice.^12^, ^13^ The development of an H9N2 influenza VLP vaccine effective in BALB/c mice requires the expression of only three viral proteins, namely HA, NA, and M1.^14^ Thus, VLPs which resemble infectious virus particles with respect to structure and morphology have been suggested as novel vaccine candidates against various viral infections.^15^

Live poultry markets and farms in Shanghai have been under surveillance since 2006; during this time, H9N2 has been detected in both poultry and swine. The co‐circulation of multiple H9N2 genotypes over the past decade has led to the emergence of the G57 genotype, which displays a changed antigenicity and improved adaptability in chickens.^16^ It has become predominant in vaccinated farm chickens in China, causing widespread influenza outbreaks before the H7N9 viruses emerged in humans; the latter viruses have inherited their internal genes from G57 genotype strains. In a previous study, the immunity efficacy of the currently used vaccine (A/chicken/shanghai/F/1998) against H9N2 has been evaluated and found unable to provide complete vaccine protection.^17^ Here, the HA and NA genes from a strain belonging to G57 genotype were used to construct a potential VLP‐based vaccine to protect against H9N2.

## 2 MATERIALS AND METHODS

### 2.1 Ethics statement

The conduct of this study followed animal welfare guidelines set out by the World Organization for Animal Health and was approved by the Shanghai Municipal Commission of Agriculture (Permit number: 2013^18^).

### 2.2 Cloning of HA and NA genes

Viral RNA was extracted from a 200 μL volume of a centrifuged suspension of H9N2 virus (A/chicken/Shanghai/06/2015) using a QIAamp^®^ MinElute^®^ Virus Spin kit (Qiagen, Hilden, Germany), according to the manufacturer's instructions. Reverse transcription PCR was performed using a One‐Step RT‐PCR system (Takara Bio Inc., Dalian, China) based on gene‐specific oligonucleotide primers. Following the RT‐PCR, the resulting cDNA sequences (containing the H9N2 HA and NA genes) were cloned into the pFastBac™ dual vector (Invitrogen, Waltham, MA, USA). The HA and NA sequences were found to be identical to those deposited in GenBank (respectively, KU720440 and KU720446).

### 2.3 Generation of recombinant baculoviruses

The HA gene was represented by a 1.7‐Kbp *Xho*I‐*Kpn*I fragment, lying downstream of the AcMNPV p10 promoter within the pFastBac™ dual vector, while the NA gene was represented by a 1.4‐Kbp *Bam*HI‐*Eco*RI DNA fragment, located downstream of the AcMNPV polyhedrin promoter. The resulting plasmid harbored both HA and NA driven by different promoters. Recombinant bacmids were produced by site‐specific transposition following transformation of the plasmid harboring HA and NA into *E. coli* DH10Bac cells, which contain a baculovirus shuttle vector (bacmid) and a helper plasmid (Invitrogen). The recombinant bacmid DNA was transfected into Spodoptera frugiperda (Sf9) cells (ATCC^®^ CRL1711™, www.atcc.org) seeded in 6‐well plates at a density of 0.5 × 10^6^ cells/mL using the Cellfectin reagent (Invitrogen).

### 2.4 Production of H9N2 VLP and preparation of VLP vaccine

The rescued recombinant baculovirus was propagated and titered on Sf9 cells. Sf9 cells were plated in 6‐well plates, and 10‐fold serial dilutions of recombinant baculoviral stock were prepared. Different dilutions of baculovirus were added to Sf9 cells, and cells were infected for 1 hour. Then, the virus was removed and the cell monolayer was overlaid with plaquing medium. The cells were incubated for 7‐10 days and were stained with neutral red. The number of plaques in each dilution was counted.

Recombinant baculoviruses expressing the H9N2 proteins were amplified by infecting Sf9 cells seeded in shaker flasks at 2 × 10^5^ cells/mL with a titer of 4 × 10^5^ pfu/mL. At 72 hours postinfection, culture supernatants containing the recombinant baculoviruses were harvested, clarified by centrifugation (3000 *g*, 30 minutes), and stored at −80°C. The expression of recombinant proteins was determined by SDS‐PAGE followed by Coomassie Blue staining and by Western blotting using a H9N2‐specific serum (A/chicken/Shanghai/06/2015). The secondary antibody used was goat anti‐chicken IgY (H+L) conjugated with horseradish peroxidase (Sigma, St. Louis, MO, USA).

H9N2‐specific serum (A/chicken/Shanghai/06/2015) was collected from immunized chickens. Three‐week‐old SPF white leghorn chickens were immunized intramuscularly with the inactivated strain (A/chicken/Shanghai/06/2015). For this virus, four chickens were immunized with 10^6^EID_50_ of β‐Propiolactone ‐inactivated strain combined with Freund's adjuvant (complete for the first vaccination and incomplete for the booster). Two or three doses of inactivated strain were administered approximately 2‐3 weeks apart. When HI titers to homologous virus reached 2^8^‐2^10^, blood from the four chickens was mixed. H9N2‐specific serum (A/chicken/Shanghai/06/2015) was inactivated at 60°C for 10 minutes and stored at −30°C until use.

As the VLP antigen used was not sucrose‐purified (to reduce production costs), its antigen content was quantified in terms of hemagglutination units (HAU) as described in the OIE manual,^18^ rather than by measuring the total protein concentration.^19^

### 2.5 Electron microscopy

The culture medium containing VLPs was treated for 24 hour at 4°C with 2% glutaraldehyde in phosphate‐buffered saline (PBS) (pH 7.2), adsorbed on a freshly discharged plastic/carbon‐coated grid, which was then rinsed in deionized water (East China Normal University, Shanghai, China). The grids were finally stained with 2% sodium phosphotungstate (pH 6.5), and the VLPs were observed by transmission electron microscopy at a magnification of 6000‐100 000×.

### 2.6 Neuraminidase assay

Neuraminidase activity was determined using a Neuraminidase Assay Kit (Sigma), according to the supplier's protocol.

### 2.7 Vaccination and challenge

A group of 15 three‐week‐old SPF chickens was divided into three subgroups of five chickens each. Two weeks postprime, chickens primed via intramuscular (i.m.) immunization were boosted with 0.2 mL immunogen. One subgroup received a commercial H9N2 whole‐virus‐inactivated vaccine (A/chicken/Shanghai/F/1998 strain), containing 2^12^ HAU titer of inactivated allantoic fluid, according to the manufacturer's instructions. The second subgroup received the VLP vaccine, which was prepared by emulsifying culture supernatant at an HAU titer of 2^6^ with Freund's adjuvant (complete for the first vaccination and incomplete for the booster); the final group received an emulsion of PBS which used the same adjuvant as VLP vaccine. One week after the booster injection, each group comprising of five chickens with different HI antibody titers was challenged intranasally with 10^6^ EID_50_ of H9N2 (A/chicken/Shanghai/06/2015). Tracheal and cloacal swabs were collected from each chicken to determine the extent of replication of the influenza virus, at 1, 2, 3, 5, and 7 days postchallenge (dpc), respectively. The virus shedding was quantified by the 50% egg infectious dose (EID_50_), and EID_50_ was determined by the Reed and Muench method.^20^ In order to determine immunogenicity, serum samples were collected prior to both the first and the boost vaccination and one week after the boost vaccination, and subjected to an OIE standard HI method using formalin‐inactivated antigen (A/chicken/Shanghai/10/2001 and A/chicken/Shanghai/06/2015).

### 2.8 Statistical analysis

Statistical comparisons between vaccinated and nonvaccinated chickens were performed using Fisher's exact test. An analysis of variance using the Tukey‐Kramer post‐test was used to compare virus shedding titers. Statistical significance was assigned to differences applying a *P*‐value threshold of .05.

## 3 RESULTS

### 3.1 Expression of VLP vaccine for influenza A/chicken/Shanghai/06/2015 (H9N2) virus

**Figure 1:**
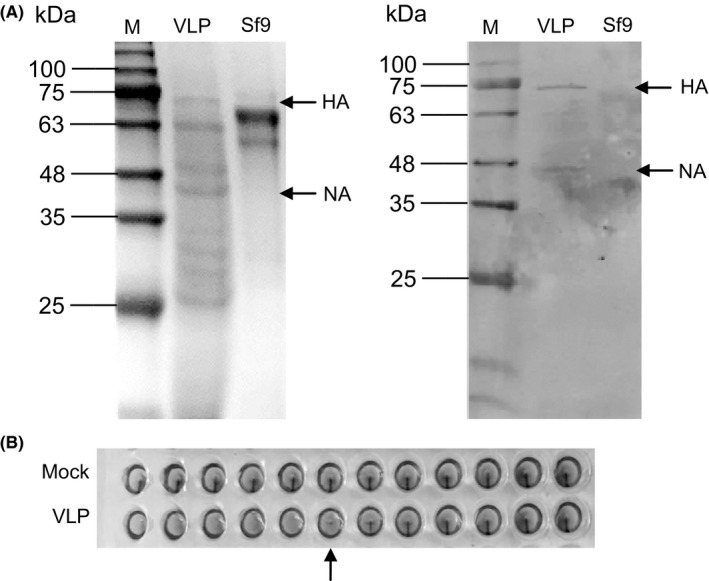
Preparation of H9N2 subtype virus‐like particle (VLPs). (A) Detection of the hemagglutinin (HA) and neuraminidase proteins within the VLPs using SDS‐PAGE and Western blotting. The influenza proteins are indicated by arrows. M: molecular weight standards. (B) VLPs harvested 72 h after transfection of Sf9 cells. The hemagglutination test was performed by exposing chicken erythrocytes to VLPs. The arrow indicates the HA titer

**Figure 2:**
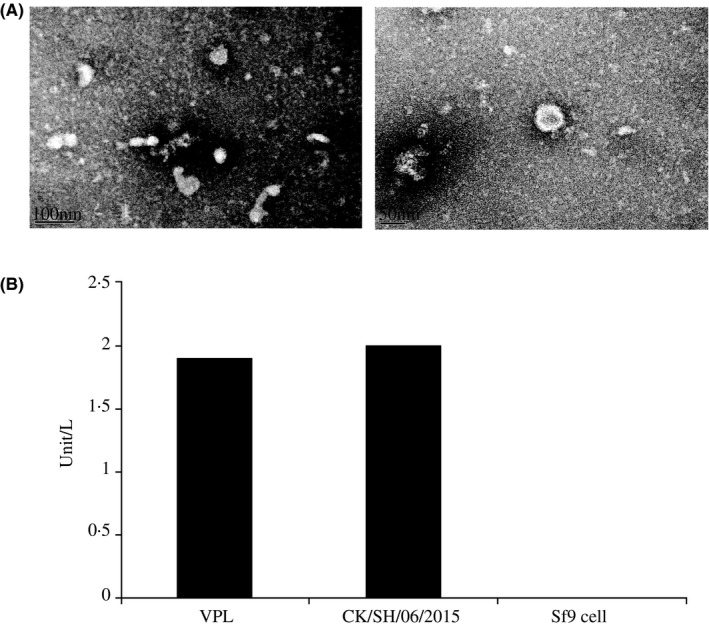
(A) Electron micrograph of a negatively stained H9N2 virus‐like particle (VLP). (B) A neuraminidase (NA) assay using either VLPs or H9N2 shows that both featured an hemagglutinin titer of 2^6^. The supernatant of Sf9 cells was used as a negative control. Neuraminidase activity was expressed as unit/L

The VLPs generated by the recombinant baculoviruses harboring the genes encoding H9N2 HA and NA proteins were tested for the presence of the influenza proteins using SDS‐PAGE and Western blotting, and also a hemagglutination and a neuraminidase assay. The HA and NA genes encode a polypeptide of, respectively, ~70 KDa and 46 KDa. The authenticity and the expected molecular weights of HA and NA proteins were confirmed by coomassie‐stained SDS‐PAGE (Figure 1A), followed by a Western blot challenged with a polyclonal serum raised against A/chicken/Shanghai/06/2015 (Figure 1B), showing that their molecular weight and immunological behavior were as expected. Through the hemagglutination assay, the VLP‐containing culture supernatant had an HAU of 2^6^. A neuraminidase assay established that the VLPs possessed functional neuraminidase activity (Figure 2B): their activity was 1.9 unit/L, compared to that of A/chicken/Shanghai/06/2015 (2.0 unit/L), while that of the Sf9 cell supernatant was zero. Examination of negatively stained samples using electron microscopy revealed that the diameter of the VLPs was ~80 nm, a bit smaller than the size of influenza virus particles, maybe because there were only two structural proteins to assemble the VLPs (Figure 2A).

### 3.2 The immune response to the H9N2 VLPs

**Figure 3:**
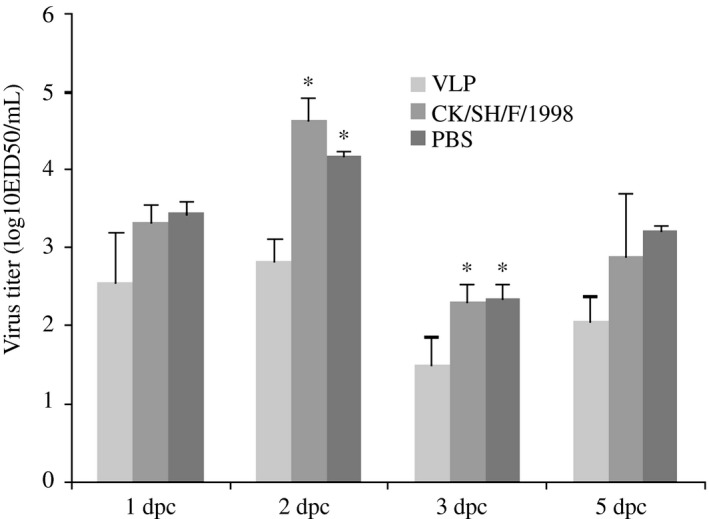
Virus titers in H9N2‐challenged chickens previously inoculated with either virus‐like particles, a commercial vaccine (A/chicken/Shanghai/F/1998 strain), or PBS. Each group of five chickens was vaccinated intramuscularly twice (separated by 14 d), then challenged intranasally with 10^6^ EID_50_ H9N2. Tracheal and cloacal swabs were collected from each chicken to determine the extent of replication of the influenza virus, at 1, 2, 3, 5, and 7 d postchallenge (dpc), respectively. The virus shedding was quantified by the 50% egg infectious dose (EID_50_), and EID_50_ was determined by the Reed and Muench method. No virus was detectable in the cloacal swabs. Virus was present in the tracheal swabs of both nonvaccinated and vaccinated chickens at 1, 2, 3, and 5 dpc. No virus was detected in all tracheal swabs at 7 dpc. (Note: Data marked *differentiated significantly, *P* < .05)

After the second (boost) vaccination, the commercial H9N2 whole‐virus‐inactivated vaccine induced antibody to H9N2 (A/chicken/Shanghai/10/2001) equivalent to 2^10^ HI units, when the titers induced by VLP vaccine lay in the range 2^4^‐2^8^. However, the VLP vaccine induced antibody to H9N2 (A/chicken/Shanghai/06/2015) equivalent to 2^10^ HI units, while the titers induced by commercial H9N2 whole‐virus‐inactivated vaccine lay in the range 2^4^‐2^8^ (Table 1). There was no detectable induction of antibody in chickens which had received a mock vaccination (PBS/adjuvant). Thus, the VLP vaccine was able to induce a better H9N2‐specific functional antibody response to the recent H9N2 strain than currently used commercial vaccine. No virus was detectable in the cloacal swabs. Virus was present in the tracheal swabs of both nonvaccinated and vaccinated chickens at 1, 2, 3, and 5 dpc (Figure 3). No virus was detected in all tracheal swabs at 7 dpc. Less viruses were shed by VLP‐immunized subgroup among the three subgroups. On 2 and 3 dpc, statistically significant differences (*P* < .05) were noted in the amount of shedding virus between VLP‐immunized subgroup and the other two subgroups, but no statistically significant differences (*P* < .05) existed among the other two subgroups. As a result, VLP vaccine can prevent the shedding of H9N2 influenza virus moderately, compared to currently used commercial vaccine.

**Table 1:** Antibody responses following immunization

Vaccine	A/chicken/Shanghai/10/2001 as HI antigen		A/chicken/Shanghai/06/2015 as HI antigen	
	14 d after the first immunization (average titer)	7 d after the boost (average titer)	14 d after the first immunization (average titer)	7 d after the boost (average titer)
Virus‐like particle (A/chicken/Shanghai/06/2015 (H9N2))	2^2^‐2^8^ (85.2)	2^4^‐2^8^ (142.4)	2^10^ (1024)	2^10^ (1024)
Commercial vaccine(A/chicken/Shanghai/F/1998)	2^10^ (1024)	2^10^ (1024)	2^2^‐2^8^ (141.2)	2^4^‐2^8^ (120)
Control	0	0	0	0

## 4 DISCUSSION

In China, an inactivated H9N2 subtype influenza virus vaccine (A/Chicken/Shandong/6/1996) was approved for prevention of H9N2 influenza virus infections in 1998. Since then, monovalent and combined vaccines have been seen on the market.^21^ Although there are other commercial vaccines used in poultry farms against H9N2 viruses in China, all the vaccines were inactivated virus. To note, the isolation time of these seed strains was from 1994 to 2005.^22^ However, H9N2 influenza virus is evolving by reassortment and mutation and so on. Most recently, Pu et al^16^ revealed that G57 had become the predominant H9N2 genotype in chicken population. Importantly, the G57 viruses have developed the ability to escape from immunization with changed antigenicity and improved fitness. Furthermore, our study also showed that antigenic groups typically correspond with the phylogenetic relationships between viruses and that currently used commercial vaccine provides a reduced level of protection against more recent H9N2 strains.^17^ Thus, this underlines the need for updating the vaccine used to give protection against current strains.

Although the first cell‐based and recombinant subunit influenza vaccines have been introduced and approved by the regulatory agencies, the majority of influenza vaccines are still made in eggs, and if there is an outbreak of avian influenza or other disease that affects chicken flocks, the supply of vaccine could be threatened.^23^, ^24^ Moreover, cost is also one of the major impediments to the more widespread use of vaccination in poultry production. An advantage of the VLP approach is that it can respond rapidly to the development of new strains of the virus. It was reported that influenza virus hemagglutinin (HA) and neuraminidase, but not the matrix protein, are required for assembly and budding of plasmid‐derived VLPs.^25^ In our study, we chose HA and NA to construct VLP. Consistent with reported data, our results demonstrated that the formation of VLP was alike to the influenza virus particles under electron microscopy. Other studies suggested that detected broader reactivity in baculovirus‐derived VLP vaccines containing M1.^26^, ^27^, ^28^ The protection of VLP including HA, NA, and M1 needs to be further investigated.

Furthermore, the supernatant, without concentration, which had an HA titer of 2^6^ generated by VLPs, is sufficient to induce a higher HI antibody in chickens against the donor strain than the currently used commercial vaccine. Regarding on our analysis, A/chicken/Shanghai/F/1998 and A/chicken/Shanghai/10/2001 were similar on genetic and antigenic levels. Yet, the virus strain of A/chicken/Shanghai/F/1998 was not available in our laboratory. Thus, we chose A/chicken/Shanghai/10/2001 (Harbin veterinary research institute, CAAS) as standard antigen in HI test. Interestingly, although there were higher HI antibody titers induced by VLP vaccine co‐administered with Freund's adjuvant via intramuscular injection, it was unable to prevent virus shedding in trachea after the chickens had been challenged. In contrast, virus shedding (irrespective of the groups) in cloacal swabs was not observed in chickens infected with H9N2 virus. Consistent with our results, it has been shown that VLP could not fully prevent the shedding of A/chicken/Shanghai/06/2015 belonging to G57 genotype.^16^ Given that the baculovirus‐derived VLPs have been shown to induce an IgA antibody‐type response in lung with intranasal (i.n) vaccination,^29^ the future investigation will be determined by intranasal vaccination of VLPs. Furthermore, it is also possible that optimization of adjuvant could raise the level of induced humoral, cellular, and mucosal activity.

It is important that VLPs for human use have to be resorted by sucrose density gradient purification,^19^, ^30^, ^31^, ^32^ a process of which is both capital intensive and time‐consuming. In comparison, for veterinary use, VLPs containing active baculovirus promote innate immune response at the site of inoculation, which may be responsible for the enhanced immunogenicity of VLP vaccination in poultry.^33^ According to this character, our baculovirus‐expressed influenza VLP antigen was elaborated without extensive purification: all that was required was to include a low‐speed centrifugation step in order to remove large cell debris. To note, our results showed that no inflammation or other adverse events were observed at the sites of injection in our research. In summary, these results indicate that nonreplicating influenza VLPs represent a promising strategy for the development of a safe and effective vaccine to prevent and control H9N2 influenza viruses.

## CONFLICT OF INTEREST

The author declares no competing interests.
